# Antitumor effects of the antiparasitic agent ivermectin via inhibition of Yes-associated protein 1 expression in gastric cancer

**DOI:** 10.18632/oncotarget.22587

**Published:** 2017-11-21

**Authors:** Sho Nambara, Takaaki Masuda, Miki Nishio, Shotaro Kuramitsu, Taro Tobo, Yushi Ogawa, Qingjiang Hu, Tomohiro Iguchi, Yousuke Kuroda, Shuhei Ito, Hidetoshi Eguchi, Keishi Sugimachi, Hiroshi Saeki, Eiji Oki, Yoshihiko Maehara, Akira Suzuki, Koshi Mimori

**Affiliations:** ^1^ Department of Surgery, Kyushu University Beppu Hospital, Beppu, Oita 874-0838, Japan; ^2^ Medical Institute of Bioregulation, Graduate School of Medical Sciences, Kyushu University, Fukuoka 812-8582, Japan; ^3^ Division of Molecular and Cellular Biology, Kobe University Graduate School of Medicine, Chuo-Ku, Kobe, Hyogo 650-0017, Japan; ^4^ Department of Pathology, Kyushu University Beppu Hospital, Beppu, Oita 874-0838, Japan; ^5^ Digestive Disease Center, Showa University Northern Yokohama Hospital, Tsuzuki-Ku, Kanagawa 224-8503, Japan; ^6^ Department of Gastroenterological Surgery, National Kyushu Cancer Center, Fukuoka 811-1395, Japan; ^7^ Department of Surgery and Science, Graduate School of Medical Sciences, Kyushu University, Fukuoka 812-8582, Japan

**Keywords:** ivermectin, yes-associated protein 1 inhibitor, gastric cancer, antiproliferative effect, therapeutic target

## Abstract

Yes-associated protein 1 (YAP1) acts as an oncogene through dephosphorylation and nuclear translocation, and nuclear accumulation of YAP1 is associated with poor prognosis in gastric cancer (GC). We previously identified ivermectin, an antiparasitic drug, as a YAP1 inhibitor. Here, we aimed to clarify whether ivermectin had antitumor effects on GC through inhibition of YAP1. First, we evaluated the antiproliferative effects of ivermectin on human GC cells using *in vitro* proliferation assays and a xenograft mouse model. YAP1-knockdown assays were performed to assess whether the sensitivity to ivermectin depended on YAP1 expression. Next, we explored the mechanism through which ivermectin regulated YAP1 expression or localization by immunoblotting and reverse transcription-quantitative polymerase chain reaction for YAP1 and the downstream gene *CTGF*. Finally, the clinical significance of *YAP1* expression was examined using three independent GC datasets. We found that MKN1 GC cells were most sensitive to ivermectin, whereas MKN7 cells were most resistant. In MKN1 xenografts, ivermectin suppressed tumor growth, and the sensitivity of MKN1 cells to ivermectin was decreased by YAP1 knockdown. Ivermectin inhibited YAP1 nuclear expression and CTGF expression in MKN1 cells but not MKN7 cells. Moreover, ivermectin decreased *YAP1* mRNA expression, thereby inhibiting nuclear accumulation of YAP1 in MKN1 cells. In survival analysis, low *YAP1* mRNA expression was associated with a better prognosis in three independent GC datasets. In conclusion, we identified ivermectin as a potential antitumor agent and found a promising novel therapeutic strategy for inhibition of GC progression by blocking YAP1 expression.

## INTRODUCTION

Gastric cancer (GC) is the fifth most common malignancy and the third leading cause of cancer-related death worldwide [1]. Despite recent advances in medical treatments, such as chemotherapy and biological therapy, for the management of GC, patient survival remains poor, particularly for those with advanced disease [2], highlighting the need for the development of novel therapeutic agents.

Yes-associated protein 1 (YAP1) is upregulated and exhibits oncogenic properties in GC. Moreover, increased nuclear expression of YAP1 is associated with poor prognosis in patients with GC [3, 4], colon cancer, ovarian cancer, and lung cancer [5–7]. YAP1 is a downstream target of the Hippo signaling pathway, which regulates organ size during development [8]. YAP1 acts as a transcriptional co-activator in the nucleus, activating TEA domain transcription factor (TEAD)-mediated transcription of cell proliferation genes, such as connective tissue growth factor (CTGF) [9]. Activation of the Hippo pathway phosphorylates YAP1 at Ser127, which inhibits the activity of YAP1 and results in retention of YAP1 in the cytoplasm [10, 11]. Thus, nuclear YAP1 is a positive regulator of cell proliferation that is suppressed by Hippo signaling. Hence, inhibition of YAP1 expression may prevent tumor progression and improve prognosis in various malignancies, including GC [3–7].

We previously identified ivermectin as a potential YAP1 inhibitor by chemical compounds screening [12]. Ivermectin is a chemically modified derivative of avermectin, which was initially purified by Drs. Campbell and Omura as an effective antiparasitic agent [13, 14], earning these researchers a Nobel Prize in Physiology or Medicine in 2015. Recently, ivermectin has been reported to be a promising antitumor agent for various types of malignant tumors, including colon cancer, ovarian cancer, melanoma, and leukemia [15–18]. However, little is known about the molecular mechanisms underlying ivermectin-mediated suppression of tumor growth, and no studies have evaluated whether ivermectin has antitumor effects in GC. Accordingly, such studies may enable ivermectin to be repositioned as a novel anticancer drug.

In this study, we aimed to clarify the antitumor effects of ivermectin in GC and evaluate the mechanisms through which regulation of YAP1 expression modulates these antitumor effects.

## RESULTS

### Ivermectin suppressed GC growth *in vitro* and in a xenograft mouse model

**Figure 1:**
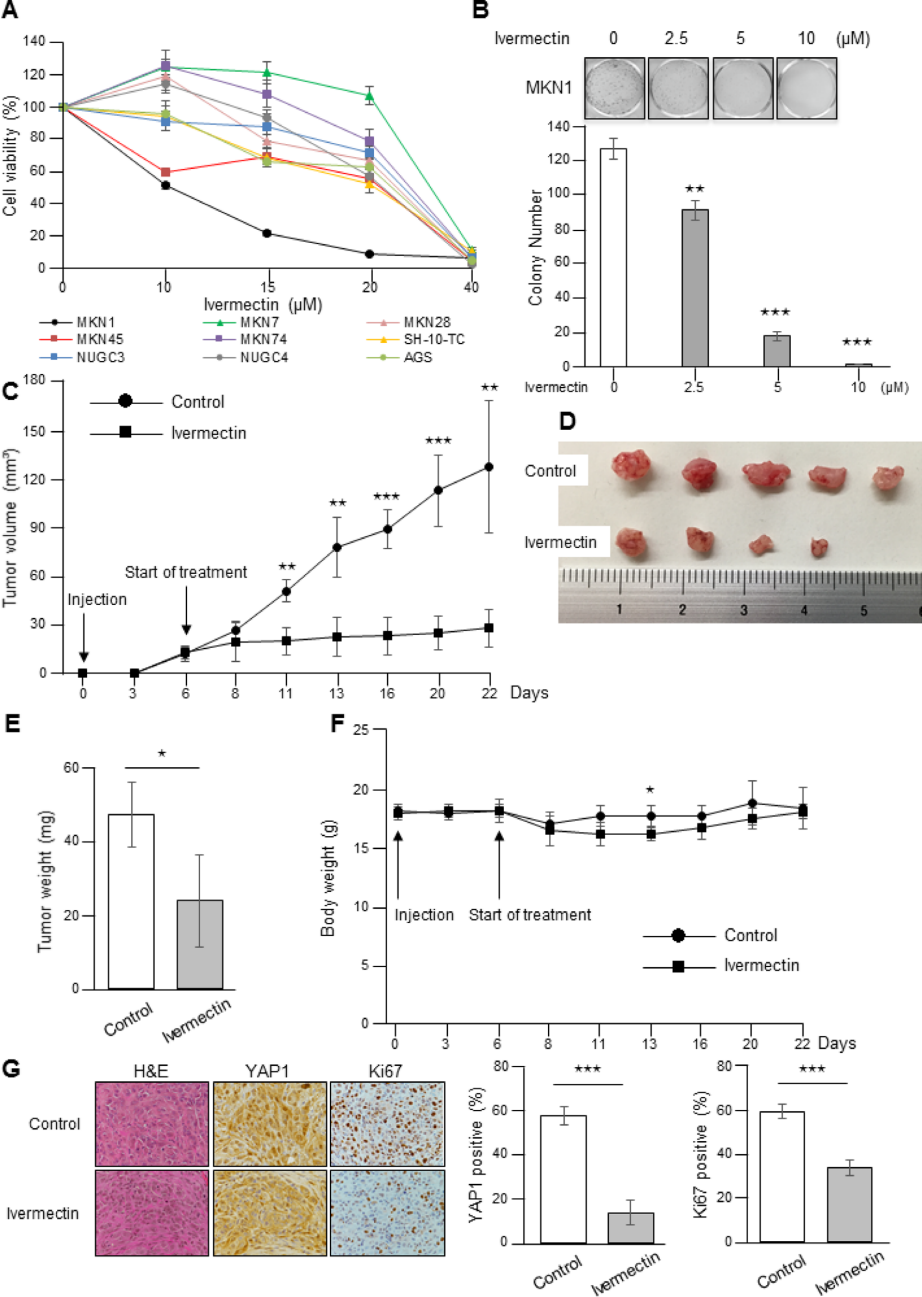
Ivermectin suppressed the growth of GC cells *in vitro* and in a xenograft mouse model (**A**) Sensitivity of GC cells to ivermectin. Cell viability was measured by MTT assays in GC cells treated with the indicated concentrations of ivermectin for 48 h. (**B**) Top, Colony formation assays. Cells were cultured with the indicated concentrations of ivermectin for 10 days. Bottom, Total number of colonies. ^**^P < 0.005, ^***^P < 0.0005. (**C**–**G**) Nude mice were inoculated with MKN1 cells and treated with ivermectin or control. (C) MKN1 tumor growth in mice treated with ivermectin or control. ^**^P < 0.005, ^***^P < 0.0005. (D) Subcutaneous tumors from control and ivermectin-treated mice. (E) Tumor weights at time of sacrifice. ^*^P < 0.05. (F) Body weights of control and ivermectin-treated mice during the entire experimental period (22 days). ^*^P < 0.05. (G) Left, Immunohistochemical staining for YAP1 and Ki67 in tumor tissues from control and ivermectin-treated mice. Original magnification, ×400. Right, Data obtained by counting nuclear YAP1- and Ki67-positive tumor cells/tumor cells per fields. ^***^P < 0.0005.

First, the antiproliferative effects of ivermectin on GC were examined by MTT assays and colony formation assays using GC cell lines. MTT assays demonstrated that the response to ivermectin was different among cell lines. Among the tested cell lines, we found that MKN1 cells were most sensitive to ivermectin (IC_50_ = 10.2 μM) and that SH-10-TC cells were also sensitive to the drug (IC_50_ = 21.2 μM; Figure 1A). Colony formation assays showed that ivermectin treatment significantly reduced colony formation in MKN1 cells (Figure 1B). In contrast, MKN7 cells were resistant to ivermectin (IC_50_ = 31.9 μM), as were MKN28 cells (IC_50_ = 25.4 μM; Figure 1A). Therefore, we defined MKN1 and SH-10-TC cells as ivermectin-sensitive cells and MKN7 and MKN28 cells as ivermectin-resistant cells. In addition, we performed apoptosis assays to determine whether ivermectin induced apoptosis in GC cells and found that ivermectin treatment did not induce apoptosis *in vitro* (Supplementary Figure 1A).

To evaluate the effects of ivermectin on GC cell growth *in vivo*, we employed a xenograft mouse model by injecting ivermectin-sensitive MKN1 cells subcutaneously into nude mice. As shown in Figure 1C, ivermectin treatment dramatically reduced tumor growth in mice. Macroscopically, ivermectin-treated tumors were much smaller than control tumors (Figure 1D). Consistent with these findings, tumor weight was reduced in ivermectin-treated mice compared with that in the control group (Figure 1E). In addition, we also assessed whether mice showed any alterations in whole body weights after ivermectin treatment. The body weights of mice from the ivermectin-treated group were similar to those of control mice during the entire treatment period, except for 7 days after the start of treatment (Figure 1F). Finally, immunohistochemical analysis for xenografts tumor tissues showed that ivermectin-treated xenografts displayed weaker YAP1 and Ki67 staining than control mice (Figure 1G). Moreover, ivermectin treatment did not induce apoptosis in tumor tissues, as demonstrated by TUNEL assays (Supplementary Figure 1B). We attempted to establish a xenograft mouse model with subcutaneous tumors using SH-10-TC. However, we were unable to establish such a model. Taken together, these data suggested that ivermectin suppressed the growth of GC *in vitro* and *in vivo* by inhibiting proliferation rather than inducing apoptosis.

### The antiproliferative effects of ivermectin were dependent on YAP1 expression

**Figure 2:**
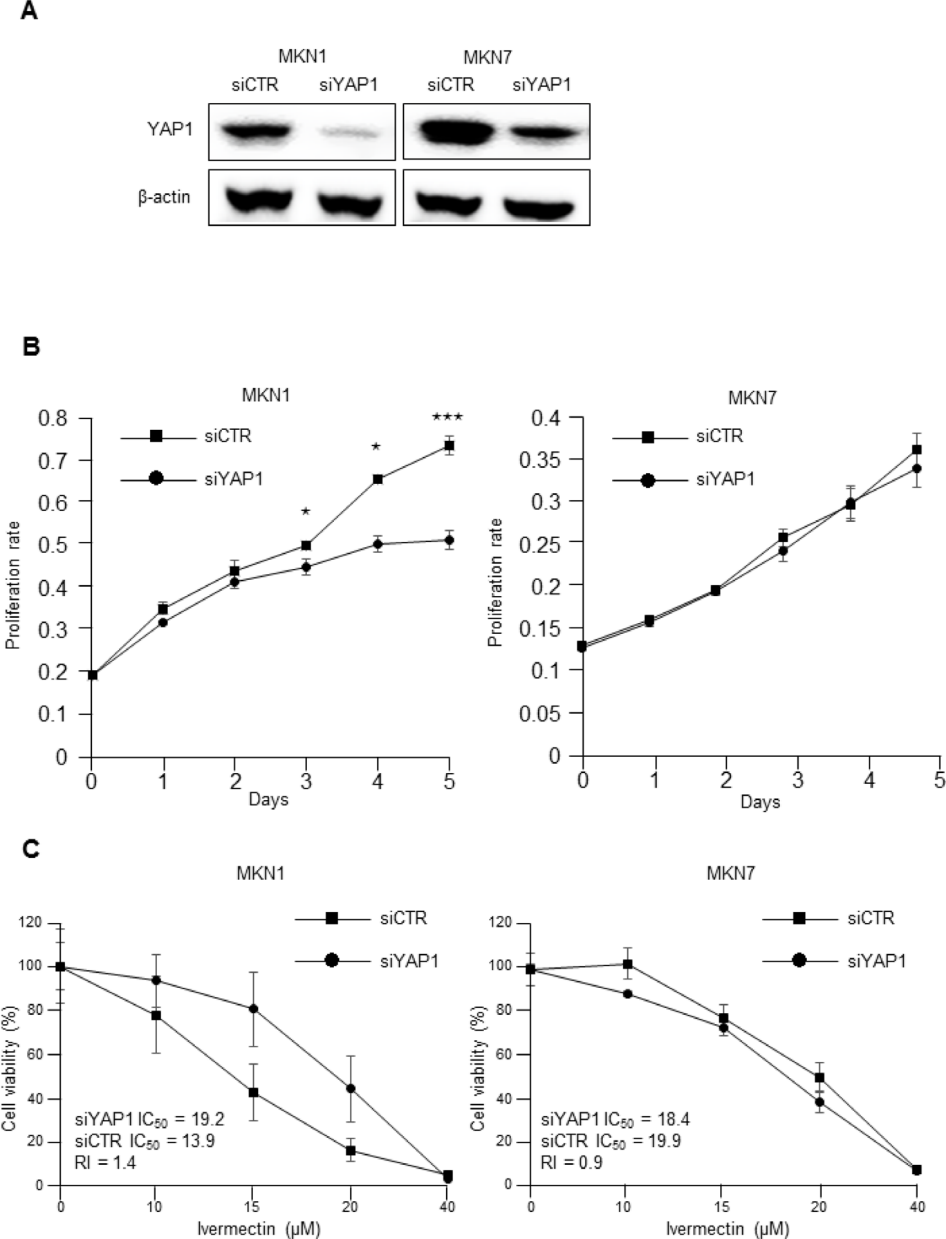
The antiproliferative effects of ivermectin were dependent on YAP1 expression (**A**) Immunoblotting for total protein expression of YAP1 in *YAP1* siRNA-transfected MKN1 and MKN7 cells and control siRNA-transfected cells. (**B**) MTT proliferation assays. The proliferation rates of *YAP1* siRNA-transfected MKN1 and MKN7 cells were compared with that of control siRNA-transfected cells. ^*^P < 0.05, ^***^P < 0.0005. (**C**) IC_50_ values and RIs for ivermectin in *YAP1* siRNA-transfected MKN1 and MKN7 cells and control siRNA-transfected cells. RI; resistance index.

MTT assays were then conducted to investigate whether the proliferation of MKN1 cells (ivermectin-sensitive) and MKN7 cells (ivermectin-resistant) was dependent on YAP1 expression. *YAP1* knockdown induced significant downregulation of YAP1 protein expression in both MKN1 and MKN7 (Figure 2A). *YAP1* knockdown significantly suppressed cell proliferation in MKN1 cells but not in MKN7 cells (Figure 2B). Thus, MKN1 cell proliferation was dependent on YAP1 expression, whereas MKN7 cell proliferation was independent of YAP1 expression. Next, we examined whether the effects of ivermectin on cell proliferation were dependent on YAP1 expression in YAP1-knockdown GC cells since ivermectin had previously been shown to inhibit YAP1 [12]. As shown in Figure 2C, the sensitivity of MKN1 cells to ivermectin was significantly decreased (siYAP1 IC_50_ = 19.2 μM, siCTR IC_50_ = 13.9 μM, RI = 1.4). This result was reproducible in another ivermectin-sensitive cell line, SH-10-TC (Supplementary Figure 2). In contrast, the sensitivity of MKN7 cells to ivermectin did not change (siYAP1 IC_50_ = 18.4 μM, siCTR IC_50_ = 19.9 μM, RI = 0.9). These data suggested that the antiproliferative effects of ivermectin depended on the expression level of YAP1 in GC cells.

### Ivermectin inhibited the nuclear accumulation of YAP1 in GC cells

**Figure 3:**
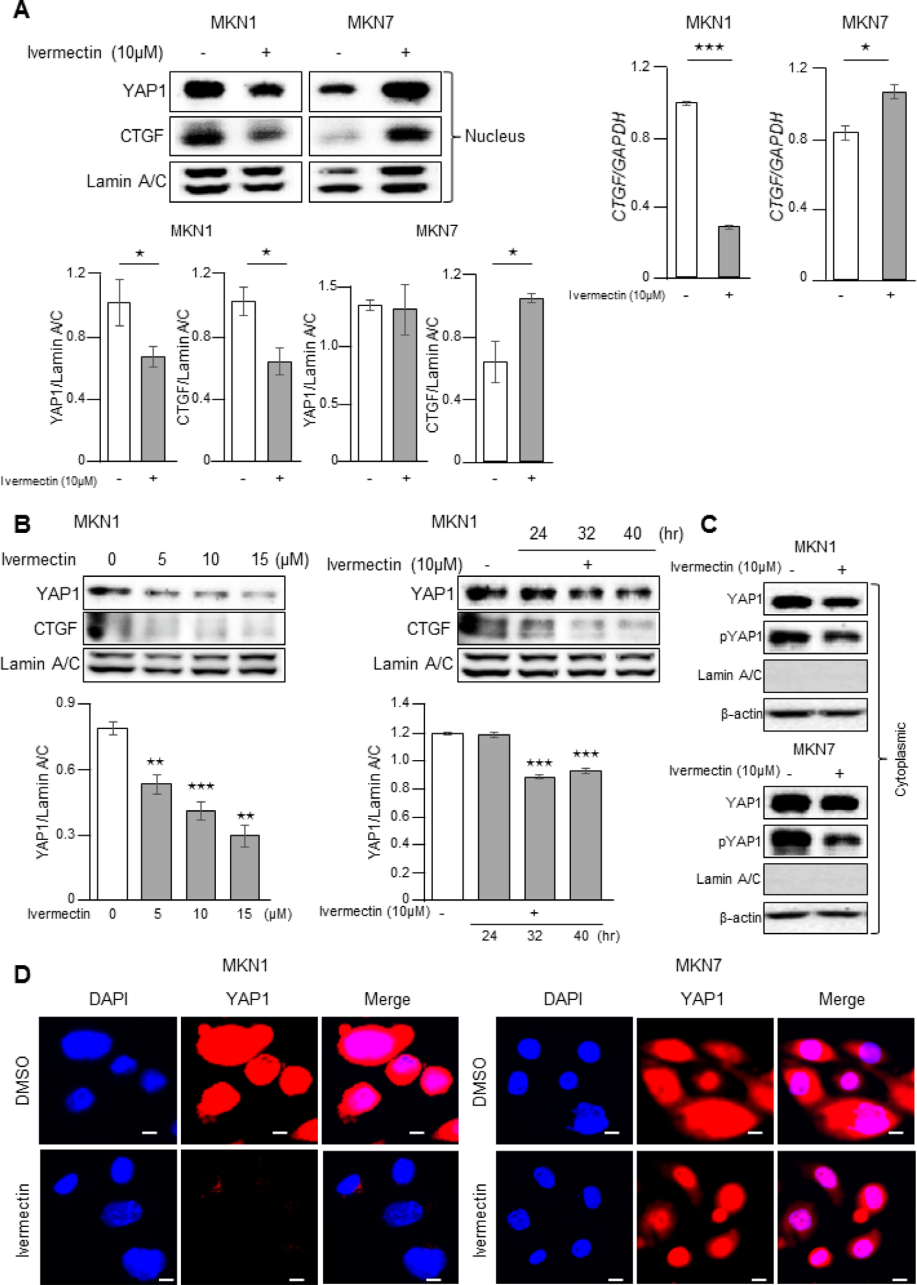
Ivermectin inhibited the nuclear accumulation of YAP1 *in vitro* (**A**, **C**, **D**) Cells were treated with vehicle (DMSO) or 10 μM ivermectin for 24 h. (A) Left-top, Immunoblotting for nuclear expression of YAP1 and CTGF in MKN1 and MKN7 cells. Left-bottom, Densitometry quantification of the band intensities. ^*^P < 0.05. Right, RT-qPCR of *CTGF* mRNA in MKN1 and MKN7 cells. ^*^P < 0.05, ^***^P < 0.0005. (**B**) Left-top, Immunoblotting for nuclear expression of YAP1 in MKN1 cells treated with the indicated concentrations of ivermectin for 24 h. Right-top, Immunoblotting for nuclear expression of YAP1 in MKN1 cells treated with 10 μM ivermectin for the indicated times. Bottom, Densitometry quantification of the band intensities. ^**^P < 0.005, ^***^P < 0.0005. (C) Immunoblotting for cytoplasmic YAP1 and phospho-YAP1 protein in MKN1 and MKN7 cells. (D) Immunofluorescence staining for YAP1 (red) in MKN1 and MKN7 cells. Scale bars, 20 μm.

Next, we investigated whether ivermectin could regulate the nuclear accumulation of YAP1, which is important for its transcriptional functions, in GC cells. As shown in Figure 3A, ivermectin treatment decreased the nuclear expression of YAP1 and the YAP1 downstream target *CTGF* in MKN1 cells but not in MKN7 cells. Furthermore, ivermectin decreased nuclear accumulation of YAP1 of MKN1 cells in both a concentration- and time-dependent manner (Figure 3B). Moreover, ivermectin did not induce the accumulation of phosphorylated YAP1 (Ser127; this phosphorylation event inhibits YAP1 activation) in the cytoplasm both in MKN1 and MKN7 cells (Figure 3C), suggesting that ivermectin did not inhibit canonical Hippo signaling. Immunofluorescence also showed that nuclear staining of YAP1 was markedly reduced after ivermectin treatment in MKN1 cells but not in MKN7 cells (Figure 3D). Thus, these results indicated that ivermectin suppressed the nuclear accumulation of YAP1 in ivermectin-sensitive GC cells.

### Ivermectin decreased *YAP1* mRNA expression and reduced nuclear expression of YAP1

**Figure 4:**
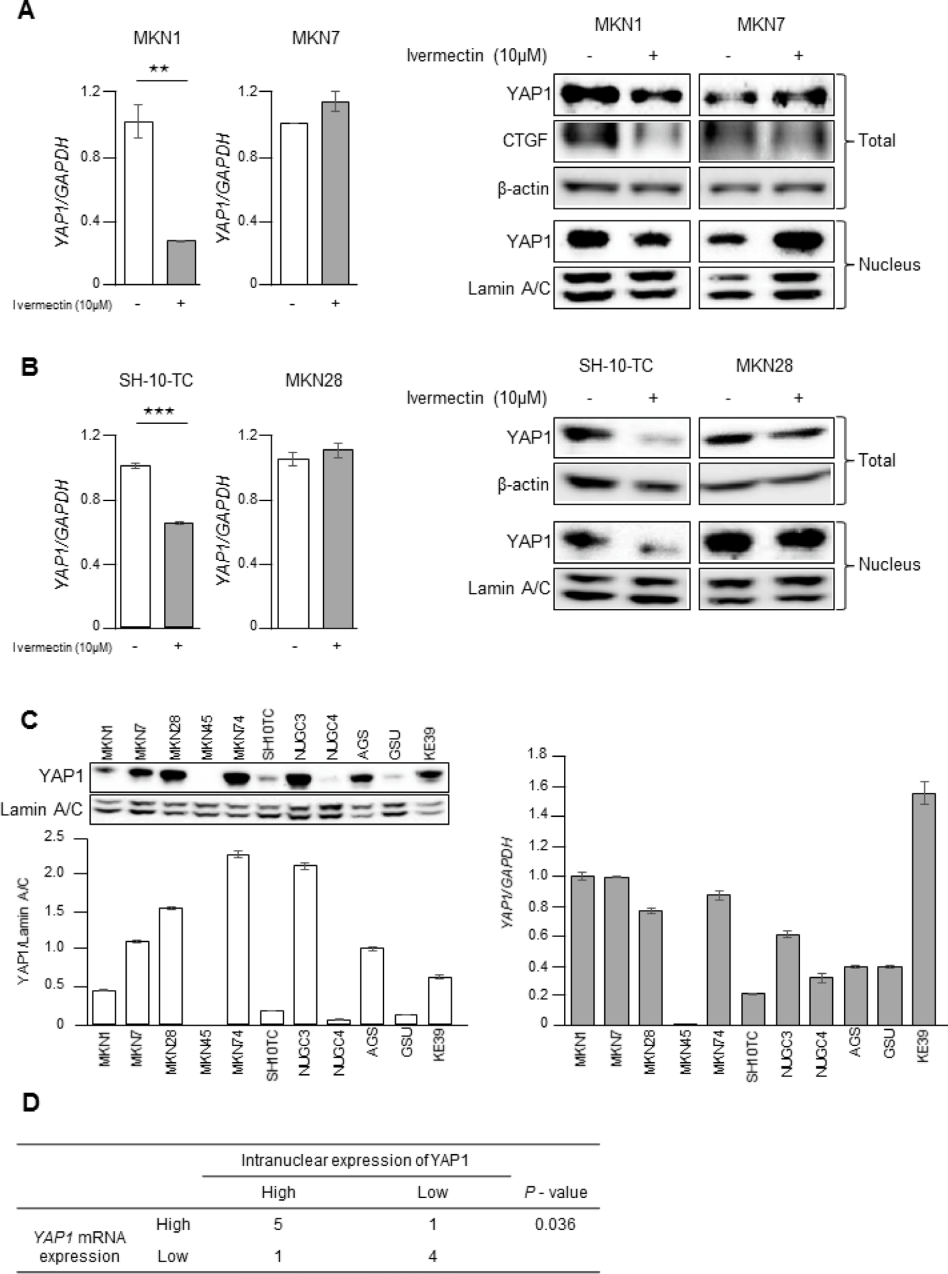
Ivermectin inhibited YAP1 expression by suppressing *YAP1* mRNA levels in GC (**A**) Left, RT-qPCR of *YAP1* mRNA in MKN1 and MKN7 cells. ^**^P < 0.005. Right, Immunoblotting for total and nuclear expression of YAP1 and CTGF in MKN1 and MKN7 cells. Cells were treated with vehicle (DMSO) or 10 μM ivermectin for 24 h. (**B**) Left, RT-qPCR of *YAP1* mRNA in SH-10-TC and MKN28 cells. ^***^P < 0.0005. Right, Immunoblotting for total and nuclear expression of YAP1 and CTGF in SH-10-TC and MKN28 cells. Cells were treated with vehicle (DMSO) or 10 μM ivermectin for 24 h. (**C**) Left-top, Immunoblotting for nuclear expression of YAP1 in 11 GC cell lines. Left-bottom, Densitometry quantification of the band intensities. Right, RT-qPCR of *YAP1* mRNA in 11 GC cell lines. (**D**) Relationship between nuclear expression of YAP1 and *YAP1* mRNA expression in GC cells.

Our experimental data showed that ivermectin suppressed the nuclear accumulation of YAP1 in GC cells. Therefore, we hypothesized that ivermectin may repress the expression of *YAP1* mRNA, thereby leading to reduced levels of nuclear YAP1. Notably, ivermectin treatment significantly decreased *YAP1* mRNA expression and whole and nuclear expression of YAP1 in MKN1 cells but not in MKN7 cells (Figure 3A and Figure 4A). This result was reproducible in other ivermectin-sensitive cells (i.e., SH-10-TC cells) and ivermectin-resistant cells (i.e., MKN28 cells; Figure 4B). Thus, ivermectin suppressed YAP1 protein expression by reducing *YAP1* mRNA expression. In order to clarify the relationship between nuclear YAP1 protein expression and *YAP1* mRNA levels, we performed immunoblotting and RT-qPCR in 11 GC cell lines (Figure 4C). There was a positive but nonsignificant correlation between nuclear YAP1 protein expression and *YAP1* mRNA levels, as demonstrated by Pearson's correlation coefficient (Supplementary Figure 3). However, there was a significant positive correlation by χ^2^ tests, as shown in Figure 4D, suggesting that nuclear expression of YAP1 protein was mildly correlated with *YAP1* mRNA levels.

### Low expression of *YAP1* mRNA in tumor tissues predicted good prognosis in patients with GC

**Figure 5:**
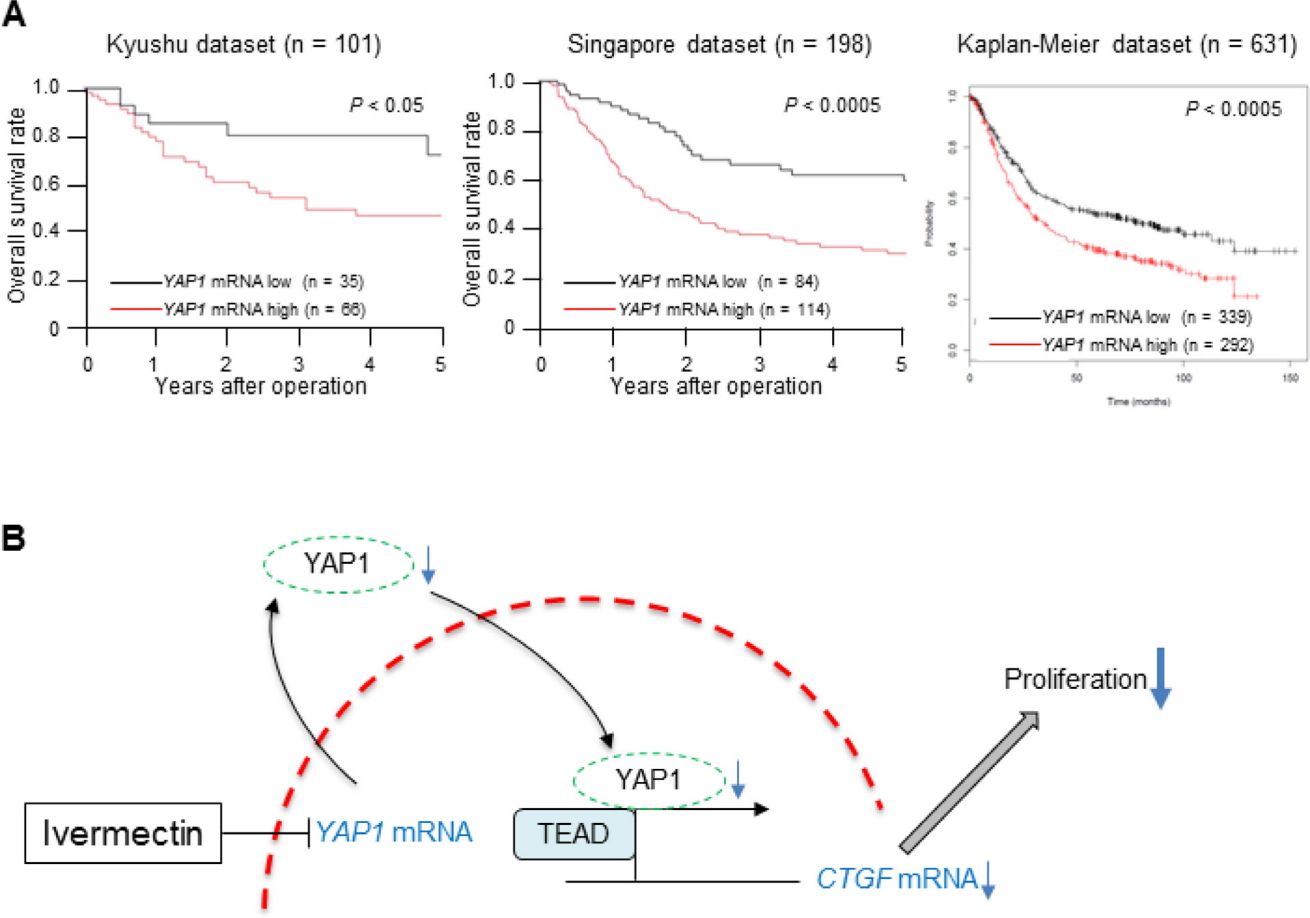
Low expression of *YAP1* mRNA in tumor tissues predicted good prognosis in GC (**A**) Overall survival rate of patients with GC according to *YAP1* mRNA expression in tumor tissues in three independent datasets (left: Kyushu dataset, middle: Singapore dataset, right: Kaplan-Meier dataset). (**B**) A proposed model showing how ivermectin inhibits YAP1 expression and suppresses tumor growth in GC.

Because the positive correlation between nuclear expression of YAP1 protein and the expression of *YAP1* mRNA suggested that *YAP1* mRNA levels could be associated with tumor aggressiveness in GC, we assessed the clinical significance of *YAP1* mRNA expression in GC. First, we examined the association between *YAP1* mRNA expression and clinicopathological factors in the Kyushu dataset (Table 1). The low *YAP1* mRNA expression group (*n* = 35) had a lower frequency of tissues showing poorly differentiated histology (*P* < 0.05), less tumor invasion (*P* < 0.05), and lower rates of venous invasion (*P* < 0.05) compared with the high expression group (*n* = 66) in the Kyushu dataset. Next, we evaluated the survival rates in three independent datasets of GC. These analyses showed that low *YAP1* mRNA expression was associated with a better prognosis than high *YAP1* expression in all three GC datasets (Kyushu dataset: *P* < 0.05, Singapore dataset: *P* < 0.0005, Kaplan-Meier dataset: *P* < 0.0005; Figure 5A). These clinical findings imply that downregulation of *YAP1* mRNA could reduce the malignant characteristics of GC, supporting our experimental results.

**Table 1:** Correlation between *YAP1* mRNA expression and clinicopathological factors of GC in the Kyushu dataset

Factors	Low (*n* = 35) Number (%)	High (*n* = 66) Number (%)	*P*-value
Age (years)			
<65	16 (45.7)	31 (47.0)	0.904
≥65	19 (54.3)	35 (53.0)	
Sex			
Male	25 (71.4)	43 (65.1)	0.522
Female	10 (28.6)	23 (34.9)	
Histology			
well/moderate	25 (71.4)	29 (43.9)	0.008^*^
poorly	10 (28.6)	37 (56.1)	
Depth of invasion			
≤SM	13 (37.1)	12 (18.2)	0.036^*^
≥MP	22 (62.9)	54 (81.8)	
Lymph node metastasis			
Absent	14 (40.0)	23 (34.9)	0.609
Present	21 (60.0)	43 (65.1)	
Lymphatic invasion			
Absent	17 (48.6)	22 (33.3)	0.134
Present	18 (51.4)	44 (66.7)	
Venous invasion			
Absent	30 (85.7)	43 (65.1)	0.028^*^
Present	5 (14.3)	23 (34.9)	
Peritoneal metastasis			
Absent	31 (88.6)	56 (84.8)	0.606
Present	4 (11.4)	10 (15.2)	
Liver metastasis			
Absent	33 (94.3)	61 (92.4)	0.726
Present	2 (5.7)	5 (7.6)	
UICC TNM Stage			
I, II	22 (62.9)	36 (54.6)	0.421
III, IV	13 (37.1)	30 (45.4)	

SM: submucosa.

MP: muscularis propria.

UICC: Union for International Cancer Control.

TNM: tumor/node/metastasis.

^*^P < 0.05.

## DISCUSSION

This is the first study to assess the antitumor effects of ivermectin, a well-known antiparasitic drug, in GC. Notably, ivermectin suppressed the proliferation of GC cells *in vitro* by decreasing nuclear expression of YAP1 in a concentration- and time-dependent manner. Moreover, ivermectin exhibited strong antitumor effects in a xenograft mouse model, with almost no adverse effects. These findings indicated that ivermectin could be a promising therapeutic drug for YAP1-dependent GC.

Interestingly, our clinical analysis demonstrated that low expression of *YAP1* mRNA in tumor tissues was associated with favorable clinicopathological phenotypes and good prognosis. These findings supported clinical evidence for a therapeutic strategy to inhibit YAP1 expression by ivermectin treatment in patients with GC. Furthermore, our findings suggested that high *YAP1* mRNA expression may be a novel biomarker of poor prognosis and could be a surrogate marker for the therapeutic efficacy of ivermectin in GC.

In this study, we assessed the mechanism through which ivermectin regulates YAP1 function for the first time and demonstrated that ivermectin decreased *YAP1* mRNA expression, resulting in reduced nuclear expression of YAP1 protein in GC cells (Figure 5B). Ivermectin-induced *YAP1* mRNA downregulation was assumed to occur through transcriptional inactivation or mRNA destabilization [19]. Further experiments are required to clarify this mechanism.

In drug discovery, one successful strategy is the exploitation of established drugs that have already been approved for treatment of other diseases (i.e., drug repositioning or drug repurposing) [20, 21]. In this study, we showed that ivermectin, which is used as an antiparasitic drug and is commercially available at a low cost, could be an effective treatment option for patients with GC. Thus, we suggest that ivermectin may be repositioned as a novel drug for the treatment of YAP1-dependent GC.

In summary, ivermectin suppressed the growth of GC *in vitro* and *in vivo* by inhibiting YAP1 expression. Furthermore, GC with low *YAP1* expression had favorable clinicopathological features and a good prognosis. These findings provided insights into the antiproliferative effects of ivermectin as a YAP1 inhibitor and established a theoretical basis for preclinical evaluations of ivermectin for management of GC.

## MATERIALS AND METHODS

### Cell culture

The human GC cell lines MKN1, MKN7, MKN28, MKN45, MKN74, SH-10-TC, NUGC-3, NUGC-4, AGS, GSU, and KE-39 were purchased from RIKEN BioResource Center (Tsukuba, Japan). Cells were maintained in Roswell Park Memorial Institute (RPMI) 1640 medium containing 10% fetal bovine serum with 100 U/mL penicillin and 100 U/mL streptomycin sulfate and cultured in a humidified 5% CO_2_ incubator at 37°C.

### Total RNA extraction and reverse transcription-quantitative polymerase chain reaction (RT-qPCR)

Total RNA from cell lines and tissues was extracted by the modified AGPC method using ISOGEN (Nippon Gene, Tokyo, Japan). RT was performed using 8 μg of total RNA with M-MLV reverse transcriptase according to the manufacturer's instructions (Invitrogen, Carlsbad, CA, USA). qPCR assessments of *YAP1*, *CTGF*, and *GAPDH* were performed using LightCycler FastStart DNA Master SYBR Green I (Roche Diagnostics, Indianapolis, IN, USA) as previously described [22]. The expression levels of *YAP1* and *CTGF* mRNA were normalized by *GAPDH* mRNA as an internal control and are expressed as values relative to the expression level of the cDNA from Human Universal Reference Total RNA (Clontech, CA, USA). The primer sequences for qPCR were as follows: *YAP1*, forward 5′-CGCTCTTCAACGCCGTCA-3′ and reverse 5′-AGTACTGGCCTGTCGGGAGT-3′; *CTGF*, forward 5′-TTGGCCCAGACCCAACTATG-3′ and reverse 5′-CA GGAGGCGTTGTCATTGGT-3′; and *GAPDH*, forward, 5′-TTGGTATCGTGGAAGGACTCTA-3′ and reverse, 5′-TGTCATATTTGGCAGGTT-3′.

### Protein extraction

For total protein extraction, cells were lysed in lysis buffer (25 mM Tris-HCl [pH 7.5], 150 mM NaCl, 0.2 mM EDTA, 0.1% NP40, 5% glycerol, and proteinase inhibitor cocktail). Extraction of nuclear and cytoplasmic protein was performed as previously described [23]. Briefly, the cell pellet was resuspended in buffer A (20 mM HEPES [pH 7.6], 10 mM NaCl, 1.5 mM MgCl_2_, 0.2 mM EDTA, 1 mM DTT, 0.1% NP40, 20% glycerol, and proteinase inhibitor cocktail), allowed to swell on ice for 10 min, and then centrifuged to collect the cytoplasmic fraction (supernatant). The nuclear pellet was resuspended in buffer B (20 mM HEPES [pH 7.6], 500 mM NaCl, 1.5 mM MgCl_2_, 0.2 mM EDTA, 1 mM DTT, 0.1% NP40, 20% glycerol, and proteinase inhibitor cocktail), incubated for 30 min on ice, and centrifuged at 15,000 rpm for 15 min to collect the nuclear fraction.

### Immunohistochemistry

Xenograft tumor tissues were fixed in 10% formalin immediately after collection. Five-micron-thick sections were cut and stained with hematoxylin and eosin (H&E), anti-YAP1 antibodies, and anti-Ki67 antibodies using the avidin-biotin-peroxidase method (LSAB2 kit; Dako, Kyoto, Japan). The primary antibodies against YAP1 and Ki67 were used at dilutions of 1:200 and 1:1000, respectively, and were purchased from Sigma-Aldrich (St. Louis, MO, USA) and Abcam (Cambridge, MA, USA), respectively. Tumor histology was independently reviewed by an experienced research pathologist at Kyushu University.

### Immunoblotting analysis

Immunoblotting analysis was performed as previously described [24]. Briefly, equal amounts of protein (35 μg) were electrophoresed on 4–20% Tris-glycine gels and then electroblotted onto Immobilon-P Transfer Membranes (Merck Millipore, Billerica, MA, USA) at 70 V for 4 h at room temperature. Nonspecific binding sites were blocked with blocking buffer (Tris-buffered saline and 0.1 % Tween-20 with 5% nonfat milk powder) for 1 h at room temperature, and the blot was incubated with specific primary antibodies in blocking buffer (anti-YAP1, anti-phospho-YAP1, anti-lamin A/C, and anti-β-actin antibodies at 1:1000 dilution; anti-CTGF antibodies at 1:200 dilution) at 4°C overnight. After washing, the blots were incubated with an appropriate secondary antibody conjugated with horseradish peroxidase for 1 h at room temperature. Blots were washed again, and detection was performed using an ImageQuant LAS 4000 Mini system (GE Healthcare Japan). Rabbit polyclonal antibodies targeting YAP1, phospho-YAP1, and lamin A/C were purchased from Cell Signaling Technology (Danvers, MA, USA) and goat polyclonal antibodies targeting CTGF and mouse monoclonal antibodies targeting β-actin were purchased from Santa Cruz Biotechnology (Santa Cruz, CA, USA). Immunoblotting densitometry analysis was performed using ImageJ software (NIH, Bethesda, MD, USA) [25]. The expression levels of YAP1 and CTGF protein were normalized according to the levels of lamin A/C for nuclear extracts. Protein concentrations were quantified by Bradford protein assays.

### Immunofluorescence

Fresh cells were fixed in 4% paraformaldehyde for 15 min, followed by permeabilization with 0.3% Triton X100. Cells were blocked with 3% bovine serum albumin for 1 h and incubated with primary antibodies at 4°C overnight. Cells were then washed with PBS, incubated with fluorescence-conjugated secondary antibodies for 1 h, and stained with DAPI. Images were acquired using a fluorescent microscope (BZ-X700; KEYENCE, Tokyo, Japan). The primary antibody against YAP1, which was the same as that used in immunohistochemistry, was used at a dilution of 1:100. The secondary antibody, Alexa Fluor 555 Conjugate, was purchased from Cell Signaling Technology.

### YAP1 siRNA transfection

*YAP1*-specific siRNA (Silencer Predesigned siRNA: sense, GGUGAUACUAUCAACCAAATT and antisense, UUUGGUUGAUAGUAUCACCTG) and negative control siRNA (Silencer Negative Control 1 siRNA) were purchased from Ambion. Transfection of MKN1, SH-10-TC, and MKN7 cells (1 × 10^4^ cells/well in 24-well plates) with siRNA oligonucleotides was performed using Lipofectamine RNAiMAX (Invitrogen) according to the manufacturer's instructions.

### MTT assay

The short-term effects of ivermectin on GC cell growth and GC cell proliferation were assessed using 3-(4,5-dimethylthiazol-2-yl)-2,5-diphenyltetrazolium bromide (MTT) assays (Roche Applied Science), as previously described [26]. After incubation for 24 h, followed by ivermectin treatment or siRNA transfection, the cells were cultured for an additional 0–120 h, and the absorbance of the samples was measured.

Resistance Index (RI) = half-maximal inhibitory concentration (IC_50_) value of cells transfected with YAP1 siRNA/IC_50_ value of cells transfected with negative control siRNA.

### Colony formation assay

The long-term effects of ivermectin on GC cell growth were assessed using colony formation assays. Cells were seeded at a density of 3000 cells/well in 6-well plates and treated with the indicated concentrations of ivermectin or vehicle control. After 10 days, the colonies were stained using a Differential Quik Stain Kit (Sysmex, Kobe, Japan) according to the manufacturer's instructions. Visible colonies were photographed using a Chemiluminescence Imaging FUSION SOLO S (VILBER, Marne la Vallée, France). Colony counts were determined using ImageJ software.

### Terminal deoxynucleotidyl transferase dUTP nick-end labeling (TUNEL) assay

TUNEL staining of xenograft tumor tissues was performed using a kit according to the manufacturer's instructions (Wako Pure Chemical Industries, Osaka, Japan).

### Apoptosis assay

Apoptosis was measured by immunoblotting analysis of cleaved poly (ADP-ribose) polymerase (PARP) and procaspase3 using corresponding antibodies (Abcam, Cambridge, MA, USA) according to the manufacturer's instructions.

### Xenograft mouse model

Five-week-old female BALB/c nu/nu mice were obtained from SLC, Inc. and maintained under specific pathogen-free conditions. All animal procedures were performed in compliance with the Guidelines for the Care and Use of Experimental Animals established by the Committee for Animal Experimentation of Kyushu University. For the xenograft model, 1 × 10^6^ MKN1 and SH-10-TC cells in 150 μL serum-free medium were injected subcutaneously into the left flanks of the mice. After visual detection of tumors, mice were treated with cyclodextrin (45%)-conjugated ivermectin (10 mg/kg, i.p., daily) or cyclodextrin carrier alone (control). Tumor sizes were measured every 3–4 days with a digital caliper and calculated using the following formula: tumor volume = length × width^2^ × 0.5. Mice were euthanized for analysis at 22 days after injection.

### Patients with GC and collection of clinical samples

Primary GC samples were obtained from 101 patients who underwent surgery at Kyushu University Beppu Hospital and affiliated hospitals from 1992 to 2009 (Kyushu dataset). All patients had a histological diagnosis of GC and were followed at 3-month intervals. The median follow-up period was 2.2 years. All patients were treated in accordance with the Japanese gastric cancer treatment guidelines edited by the Japanese Gastric Cancer Association. Written informed consent was obtained from all patients, and the Institutional Review Board of our university approved this study. Sample collection was performed as previously described [24]. Data on patient age, sex, histology, tumor depth of invasion, lymph node metastasis, lymphatic invasion, venous invasion, peritoneal metastasis, liver metastasis, and clinical stage were obtained from clinical and pathological records.

### Singapore dataset analysis

We obtained *YAP1* mRNA expression and survival data for 198 available GC cases from the Singapore dataset, as previously described [27]. Gene expression array data were deposited in the Gene Expression Omnibus database under accession number GSE30601.

### Kaplan-meier plotter analysis

The Kaplan-Meier plotter (www.kmplot.com), an online database that includes gene expression and clinical data, was used to generate the Kaplan-Meier overall survival plot as previously described (Kaplan-Meier dataset) [28].

### Statistical analysis

For continuous variables, data were expressed as means ± standard deviations, and statistical analyses were performed using Student's *t* tests. Categorical variables were compared using Pearson's correlation coefficients and χ^2^ tests or Fisher's exact tests. Overall survival was estimated using the Kaplan-Meier method, and survival curves were compared using log-rank tests. Based on the levels of *YAP1* mRNA expression in the Kyushu and Singapore datasets, cases were divided into two groups by the minimum *P*-value approach, a comprehensive method to find the optimal risk separation cutoff point in continuous gene expression measurement [29]. For YAP1 expression analysis in GC cells, we divided the 11 GC cells into two groups based on the median YAP1 expression. All tests were analyzed by JMP 12 software (SAS Institute, Cary, NC, USA). Clinicopathological factors and clinical stage were classified using the tumor-node-metastasis (TNM) system of classification.

## SUPPLEMENTARY MATERIALS FIGURES


