# Supplementary material for: Antitumor effects of the antiparasitic agent ivermectin via inhibition of Yes-associated protein 1 expression in gastric cancer
Source: Oncotarget. 2017 Nov 21;8(64):107666–77. doi: 10.18632/oncotarget.22587 (PMC5746098; doi:10.18632/oncotarget.22587)
Supplement: Supplementary file 1 [file oncotarget-08-107666-s001.pdf]

Antitumor effects of the antiparasitic agent ivermectin via inhibition of Yes-associated protein 1 expression in gastric cancer

SUPPLEMENTARY MATERIALS

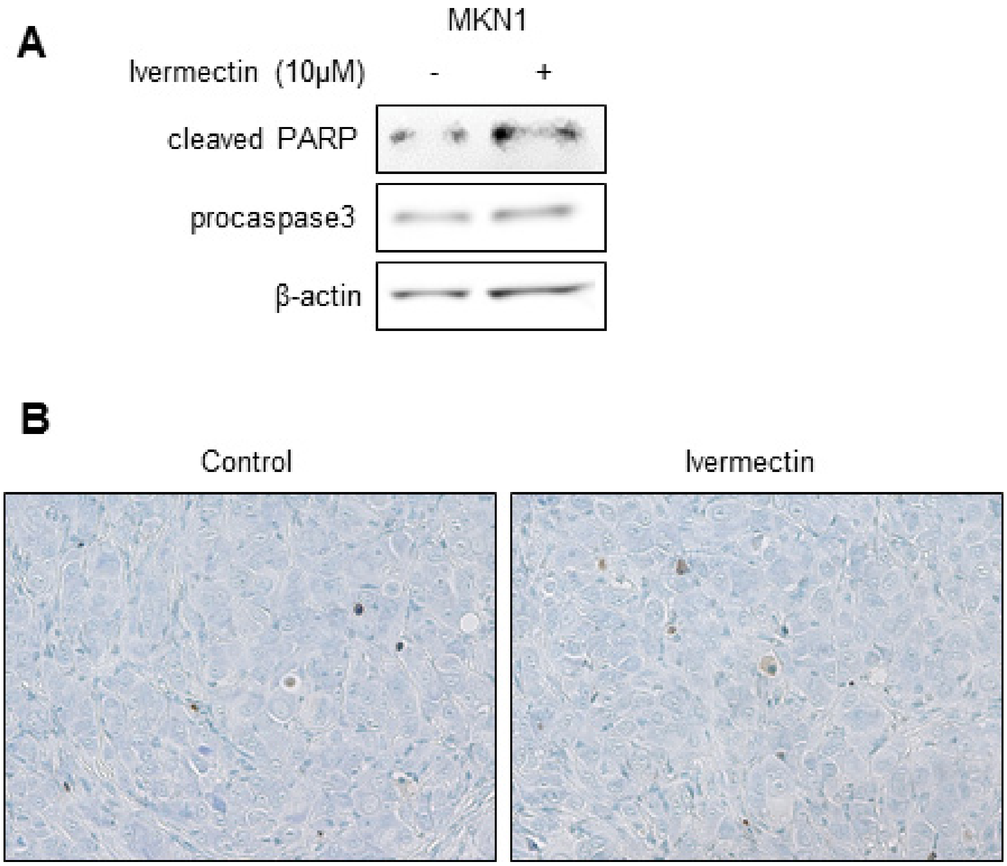

Supplementary Figure 1: Ivermectin did not induce apoptosis *in vitro* and in a xenograft mouse model. (**A**) Apoptosis assay in MKN1 cells treated with vehicle (DMSO) or 10 μM ivermectin for 24 h. (**B**) TUNEL assays in tumor tissues from control and ivermectin-treated mice. Original magnification, ×400.

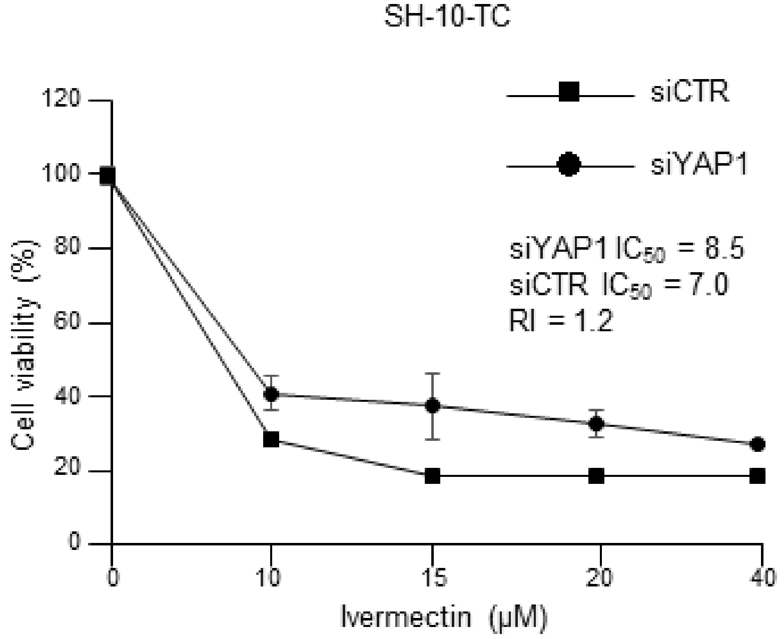

Supplementary Figure 2: The antiproliferative effects of ivermectin were dependent on YAP1 expression in ivermectin sensitive SH-10-TC cells. IC_{50} values and RI for ivermectin in *YAP1* siRNA-transfected SH-10-TC cells and control siRNA-transfected cells. RI; resistance index.

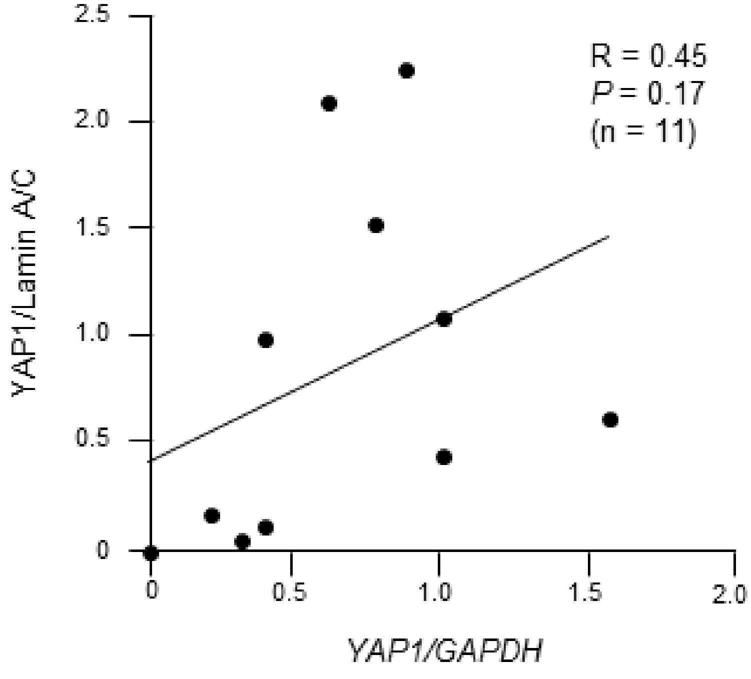

Supplementary Figure 3: There was a positive correlation between nuclear YAP1 protein expression and *YAP1* mRNA levels by Pearson's correlation coefficient. Correlation between nuclear YAP1 protein expression and *YAP1* mRNA levels in 11 GC cell lines.
